# TOM complex‐independent transport pathway of myoglobin into mitochondria in C2C12 myotubes

**DOI:** 10.14814/phy2.15632

**Published:** 2023-04-05

**Authors:** Rikuhide Koma, Tsubasa Shibaguchi, Yuhei Araiso, Tatsuya Yamada, Yudai Nonaka, Thomas Jue, Kazumi Masuda

**Affiliations:** ^1^ Graduate School of Natural Science and Technology Kanazawa University Kanazawa Ishikawa Japan; ^2^ Institute of Liberal Arts and Science Kanazawa University Kanazawa Ishikawa Japan; ^3^ Department of Clinical Laboratory Science Kanazawa University Kanazawa Ishikawa Japan; ^4^ Department of Cell Biology Johns Hopkins University School of Medicine Baltimore Maryland USA; ^5^ Department of Biochemistry and Molecular Medicine University of California Davis Davis California USA; ^6^ Faculty of Human Sciences Kanazawa University Kanazawa Ishikawa Japan

**Keywords:** skeletal muscle, Tom20, Tom40, Tom70

## Abstract

Recently, we found that myoglobin (Mb) localizes in both the cytosol and mitochondrial intermembrane space in rodent skeletal muscle. Most proteins of the intermembrane space pass through the outer mitochondrial membrane via the translocase of the outer membrane (TOM) complex. However, whether the TOM complex imports Mb remains unknown. The purpose of this study was to investigate the involvement of the TOM complex in Mb import into the mitochondria. A proteinase K protection assay of mitochondria from C2C12 myotubes confirmed that Mb integrated into the mitochondria. An immunoprecipitation assay verified the interaction of Mb and TOM complex receptors (Tom20, Tom70) in isolated mitochondria. The assay showed a clear interaction of Mb with Tom20 and Tom70. A knockdown experiment using siRNA for TOM complex receptors (Tom20, Tom70) and TOM complex channel (Tom40) did not alter the amount of Mb expression in the mitochondrial fraction. These results suggested that Mb does not necessarily require the TOM complex for mitochondrial import of Mb. Although the physiological role of Mb interactions with TOM complex receptors remains unclear, further studies are needed to clarify how Mb enters the mitochondria independently of the TOM complex.

## Graphical abstract

The purpose of this study was to investigate the involvement of the TOM complex in Mb import into the mitochondria. A knockdown experiment using siRNA for Tom40 did not alter the amount of Mb expression in the mitochondrial fraction, suggesting that Mb does not necessarily require the TOM complex for mitochondrial import of Mb.

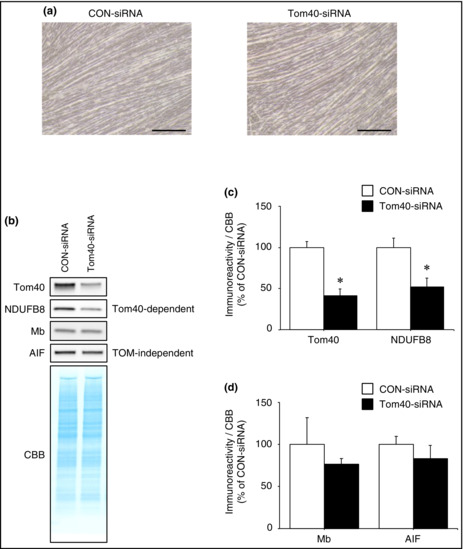

## 1 INTRODUCTION

Myoglobin (Mb) is an oxygen (O_2_)‐binding protein that belongs to the globin superfamily. Mb is highly expressed in oxidative skeletal muscle and cardiac tissue. Mb plays a principal role in O_2_ storage, buffering intracellular O_2_ concentrations and facilitating O_2_ diffusion. Although Mb is generally recognized as a cytoplasmic protein (Kanatous & Mammen, 2010; Ordway & Garry, 2004; Postnikova & Shekhovtsova, 2018), we previously demonstrated that Mb is also localized within mitochondria in rat skeletal muscle by using a proteinase K (PK) protection assay, a technique to digest proteins interacting with the outer mitochondrial membrane (OMM; Koma et al., 2021).

Our previous studies also suggested that Mb interacts with cytochrome *c* oxidase subunit IV (COX‐IV), and its overexpression augmented mitochondrial respiratory function through up‐regulation of complex IV activity in myocytes (Yamada et al., 2013, 2016). These studies demonstrated a potential role of intra‐mitochondrial Mb to up‐regulate mitochondrial respiratory function in skeletal muscle by interacting with complex IV and enhancing its activity. However, the mechanism of Mb transport into the mitochondria remains unclear.

Many proteins use the translocase of the outer mitochondrial membrane (TOM) complex as their main entry gate to the mitochondria. The TOM complex plays a critical role in translocation of proteins from the cytosol to the intermembrane space (IMS) across the OMM. The TOM complex is mainly composed of two import receptors—Tom20 and Tom70—and the pore‐forming protein Tom40. Tom20 recognizes the pre‐sequence of mitochondrial proteins, whereas Tom70 recognizes internal signal sequences in the proteins. After the recognition step by the TOM complex receptors, the protein is transferred to Tom40 and subsequently passes through the OMM (Schmidt et al., 2010; Wiedemann & Pfanner, 2017).

Although other transport mechanisms exist (Chiang et al., 2012), many mitochondrial IMS proteins are translocated through the TOM complex across the OMM (Kulawiak et al., 2013). Non‐mitochondrial proteins are also translocated into mitochondria via the TOM complex pathway (Budas et al., 2010; Fu et al., 2012; Rodriguez‐Sinovas et al., 2006). Given that Mb inside mitochondria is localized in the IMS (Koma et al., 2021), it is a possible that Mb uses the TOM complex to transport into mitochondria. The purpose of the present study was to examine whether Mb import into mitochondria is mediated by the TOM complex. Here, although we find interaction of Mb with TOM complex receptors (Tom20, Tom70), knockdown of TOM complex subunits did not alter Mb import into mitochondria of C2C12 myotubes, suggesting that the TOM complex is dispensable for Mb import into mitochondria.

## 2 MATERIALS AND METHODS

### 2.1 Cell culture and transfection

Mouse C2C12 myoblasts were obtained from American Type Culture Collection (ATCC) and seeded into 10 cm dishes in growth medium consisting of Dulbecco's modified Eagle's medium (high‐glucose DMEM; Gibco) with 10% (v/v) fetal bovine serum and 1% (v/v) penicillin–streptomycin. Cell culture and transfection experiments were performed using cells up to 16 passages. Dishes were incubated in an incubator at 37°C with 5% CO_2_. We previously confirmed that Mb expression is completely quiescent in myoblasts but increases during myotube differentiation (Yamada et al., 2016). Therefore, when the cells reached 80%–90% confluence, the medium was changed into differentiation medium (high‐glucose DMEM with 2% (v/v) calf serum, 1% (v/v) penicillin–streptomycin, and 1% (v/v) non‐essential amino acids) to induce myotube formation as well as Mb expression. Fresh differentiation medium was replaced every 24 h until differentiation day 5. The cells were fractionated into cytoplasmic and mitochondrial fractions and the quality of the mitochondrial fraction was subsequently evaluated. The mitochondrial fractions were used for PK protection assay and immunoprecipitation (IP) analysis.

Transfection experiments were performed with Lipofectamine RNAiMAX Reagent (Invitrogen) following the manufacturer's instructions and the previous studies (Chen et al., 2019; Egawa et al., 2014). The control (CON)‐siRNA (sc‐37007), Tom20‐siRNA (sc‐36692), Tom70‐siRNA (sc‐154554), and Tom40‐siRNA (sc‐61698) were purchased from Santa Cruz Biotechnology. After the myoblasts reached 40%–50% confluence, the growth medium was changed to transfection medium, which was prepared as follows: (1) siRNAs (10 μM) and Lipofectamine RNAiMAX Reagent were diluted in Opti‐MEM I Reduced Serum Medium (Gibco) to 1:11.5 and 1:16.7 ratio, respectively; (2) the diluted siRNAs and Lipofectamine RNAiMAX Reagent were mixed (1:1 ratio) and incubated at room temperature for 5 min; (3) the mixture and the growth medium were mixed well (1:39 ratio) and incubated at room temperature for 15 min (the final concentration of each siRNA was 10 nM). Following 48 h transfection, the medium was changed to differentiation medium. After 3 days of differentiation, mitochondria were isolated from some of the cells. The others were incubated for 24 h in the differentiation medium containing the Lipofectamine RNAiMAX/siRNA complexes (the final concentration of each siRNA was 10 nM). After the transfection, the medium was changed with fresh differentiation medium and myotubes were incubated in the differentiation medium for 24 h. Then, myotubes were harvested for mitochondrial isolation. The mitochondrial fractions were used for immunoblotting analysis. Images of the myotubes were captured using a DP71 digital camera (Olympus) on a light microscope (CKX41; Olympus) at 100x magnification.

### 2.2 Cell fractionation

Cell fractionation was performed according to the method of Koma et al. (2021). Briefly, the cells were homogenized in ice‐cold Solution A (250 mM sucrose, 5 mM NaN_3_, 2 mM EGTA, 20 mM HEPES‐Na, pH 7.4) using 20 strokes with a PowerMasher II (Nippi). The homogenate was then passed through a 27‐gauge syringe needle 10 times on ice. The suspension was centrifuged at 600 g for 10 min at 4°C to remove nuclei and debris. The supernatant was centrifuged at 16,000 g for 30 min at 4°C to precipitate the mitochondria. The final supernatant was used as the cytosolic fraction for immunoblotting analysis. The mitochondrial pellet was washed twice in Solution A and then re‐suspended in Solution A. The mitochondrial re‐suspension was further centrifuged at 16,000 g for 30 min at 4°C. The final pellet was then re‐suspended in Solution A and used as the mitochondrial fraction for immunoblotting analysis. The protein concentrations of the cytosolic and mitochondrial fractions were determined by the method of Bradford (1976) using a protein assay kit (Bio‐Rad Laboratories). Samples of each fraction were diluted and adjusted to a final concentration of 0.5 μg protein/μL with 2x sodium dodecyl sulfate‐polyacrylamide gel electrophoresis (SDS–PAGE) loading buffer [125 mM Tris–HCl, 4% SDS (w/v), 20% glycerol (w/v), 10% β‐mercaptoethanol (v/v), 0.002% bromophenol blue (w/v), pH 6.8] and Solution A. The samples were incubated for 5 min at 95°C before being subjected to immunoblotting analysis.

### 2.3 Proteinase K protection assay

The PK protection assay was preformed according to the method of Koma et al. (2021). Seventy‐five micrograms of mitochondrial fraction protein were incubated for 10 min on ice with a final concentration of 0 or 1 μg/mL PK, dissolved in Solution A (final volume of 500 μL). After incubation, 500 μL of 20% (w/v) trichloroacetic acid in Solution A was added to each tube and incubated on ice for 15 min to stop the PK‐induced proteolysis. All tubes were then pelleted by centrifugation at 10,000 *g* for 15 min at 4°C. The resultant mitochondrial pellets were washed twice with acetone and centrifuged at 10,000 g for 10 min at 4°C. After centrifugation, the pellets were air‐dried at room temperature for 60 min to completely remove the acetone from the pellet. The final pellets were solubilized in 150 μL of 1x SDS–PAGE loading buffer [62.5 mM Tris–HCl, 2% SDS (w/v), 10% glycerol (w/v), 5% β‐mercaptoethanol (v/v), 0.001% bromophenol blue (w/v), pH 6.8]. The samples were incubated for 5 min at 95°C before being subjected to immunoblotting analysis.

### 2.4 Immunoprecipitation analysis

IP analysis was preformed according to the modified method of Yamada et al. (2013). Fifty micrograms of mitochondrial fraction protein were added to 1 mL Dulbecco's phosphate‐buffered saline [D‐PBS (−)] aliquots in 1.5 mL tubes. Ten microliters of Dynabeads (Immunoprecipitation kit‐Dynabeads Protein G; Invitrogen, Carlsbad) were added to the samples, and the samples were rotated for 2 h at 4°C. Subsequently, the Dynabeads were collected with magnets and 1 μg of normal rabbit immunoglobulin G (IgG; #2729; Cell Signaling Technology, Danvers) was added to the supernatant and rotated overnight at 4°C. After the overnight rotation, 10 μL of Dynabeads was added to the samples, and the samples were rotated for 2 h at 4°C. The samples were then centrifuged at 7,000 g for 30 s, and the Dynabeads were collected with magnets. The resulting supernatant was rotated with 5 μg of anti‐Tom20 (11802‐1‐AP; Proteintech), anti‐Tom70 (14528‐1‐AP; Proteintech), or anti‐rabbit IgG (Cell signaling Technology) overnight at 4°C. After the overnight rotation, Dynabeads were added to the samples and incubated for 2 h at 4°C. The conjugated Dynabead‐antibody‐extracts were collected with magnets, washed with D‐PBS (−), and the immune targeted proteins were then eluted in 25 μL of 1x SDS–PAGE loading buffer. The eluates were incubated for 5 min at 95°C before being subjected to immunoblotting analysis.

### 2.5 Immunoblotting analysis

Immunoblotting analysis was performed according to the modified method of Koma et al. (2021). Equal amounts of proteins were separated by 12–15% SDS–PAGE, and the separated proteins were electrophoretically transferred onto polyvinylidene difluoride membranes (Clear Blot Membrane‐P plus; ATTO) using a semi‐dry system (WSE‐4045 HorizeBLOT 4M; ATTO). The membranes were washed with Tris‐buffered saline (150 mM NaCl, 25 mM Tris–HCl, pH 7.4) containing 0.1% (v/v) Tween‐20 (TBS‐T) for 10 min and were blocked three times with TBS‐T containing 5% (w/v) skim milk for 1 h at room temperature. After blocking, the membranes were washed three times with TBS‐T for 10 min, and then incubated with mouse monoclonal antibodies against α‐tubulin (1:20,000; 66031‐1‐Ig; Proteintech), cytochrome *c* (Cyt *c*; 1:5,000; 66264‐1‐Ig; Proteintech), oxidative phosphorylation complexes (1:1,000; ab110413; abcam), Mb (1:1,000; sc‐393020; Santa Cruz Biotechnology), Tom70 (1:5,000; 66593‐1‐Ig; Proteintech), and apoptosis‐inducing factor (AIF; 1:1,000; sc‐55519; Santa Cruz Biotechnology), and rabbit polyclonal antibodies against Tom20 (1:5,000; 11802‐1‐AP; Proteintech), voltage‐dependent anion channel (VDAC; 1:5,000; 55259‐1‐AP; Proteintech), Tom40 (1:5,000; 18409‐1‐AP; Proteintech), mitochondrial heat shock protein 70 (mtHSP70; 1:5,000; 14887‐1‐AP; Proteintech), COX‐IV (1:1,000; 11242‐1‐AP; Proteintech), and PTEN‐induced kinase 1 (PINK1; 1:1,000; 23274‐1‐AP; Proteintech) overnight at 4°C. The antibodies were diluted in TBS‐T containing 5% (w/v) bovine serum albumin and 0.02% (w/v) NaN_3_. After overnight incubation, the membranes were washed three times with TBS‐T for 10 min and reacted with horseradish peroxidase‐conjugated anti‐rabbit IgG (1:5,000; #7074; Cell Signaling Technology) or anti‐mouse IgG secondary antibody (1:5,000; #7076; Cell Signaling Technology) in TBS‐T containing 5% (w/v) skim milk for 1 h at room temperature. Following three washes with TBS‐T, protein signals were visualized by the chemiluminescence detection method using WSE‐7120 EzWestLumi plus (ATTO) or Amersham ECL select (Cytiva), and captured using a MicroChemi (Berthold Technologies).

### 2.6 Band quantification and statistical analysis

Immunoreactivities were quantified using Image J software (version 1.53a, National Institutes of Health). In the present study, membranes after protein transfer were stained with Coomassie brilliant blue (CBB) using EzStain AQua MEM (ATTO) to check for equal protein loading. The mean value for protein expression in the control group was set to 100%. All data are presented as mean ± SD. All statistical analyses were performed using SPSS Statistics 25 software (Advanced Analytics Inc.). The statistical differences were tested by unpaired *t*‐test. Significance was set at *p* < 0.05.

## 3 RESULTS

### 3.1 Cytosolic proteins do not contaminate the mitochondrial fraction

**FIGURE 1:**
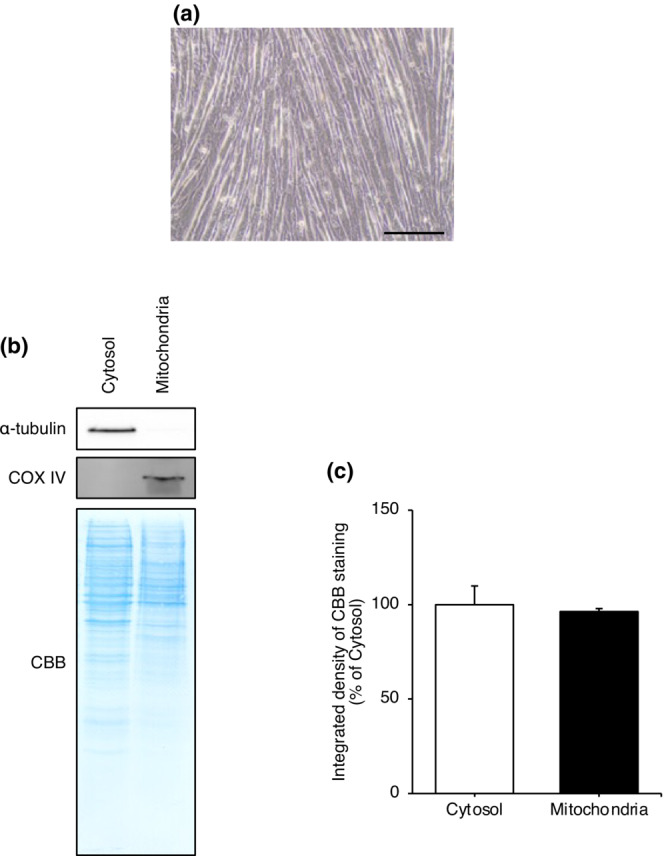
Cytosolic protein does not contaminate the mitochondrial fraction. Three independent mitochondrial isolations were performed from separate C2C12 myotubes on differentiation day 5. (a) Myoblasts were differentiated into myotubes following incubation in differentiation medium for 5 days. Scale bar shows 200 μm. (b) Cytosolic and mitochondrial fractions from C2C12 myotubes were immunoblotted for α‐tubulin (cytosolic marker protein) and COX‐IV (mitochondrial marker protein) to verify the quality of the mitochondrial fractions. CBB staining was performed to confirm equal protein loading. (c) Quantification of the integrated density of the CBB‐stained cytosolic and mitochondrial fractions (*n* = 3/group). The mean integrated density of CBB‐staining in the cytosolic fractions was set as 100%. Values are means ± standard deviation. An unpaired *t*‐test was performed to compare the values between cytosolic and mitochondrial fractions. Unprocessed blots are available in Figure S1. CBB, Coomassie brilliant blue; COX‐IV, cytochrome *c* oxidase subunit IV.

Since Mb is localized mostly in the cytoplasm (Kanatous & Mammen, 2010; Ordway & Garry, 2004), it was necessary to confirm that the mitochondrial fraction is free of cytoplasmic contamination in order to accurately assess the amount of myoglobin in the mitochondrial fraction. Therefore, the quality of the mitochondrial fraction was assessed by immunoblotting for the mitochondrial protein COX‐IV and the cytosolic protein α‐tubulin in the differentiated C2C12 myotubes (Figure 1a). There was no significant difference in total protein concentrations expressed as the integrated density of CBB between the cytosolic and mitochondrial fractions (Figure 1b,c), indicating that the total protein concentration was equal in both fractions. In this condition, COX‐IV was only detected in the mitochondrial fraction, whereas α‐tubulin was only expressed in the cytosolic fraction (Figure 1b). These data indicate that cytosolic proteins, including cytosolic Mb, did not contaminate the mitochondrial fraction used in this study.

### 3.2 Mb is localized within mitochondria of C2C12 myotubes

**FIGURE 2:**
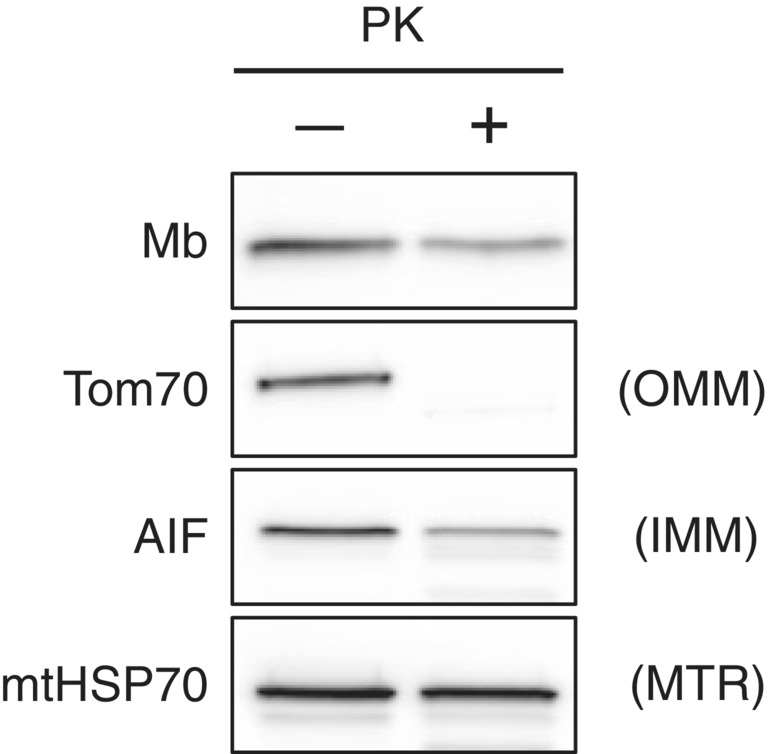
Mb is localized within the mitochondria of C2C12 myotubes.The PK protection assay was performed using mitochondria isolated from C2C12 myotubes on differentiation day 5. Immunoblotting was performed with antibodies for Mb, Tom70, AIF, and mtHSP70 on untreated mitochondria and PK‐treated mitochondria. Proteins not localized inside the mitochondria are degraded by PK‐proteolysis and are subsequently not detected. Unprocessed blots are available in Figure S2. AIF, apoptosis‐inducing factor; IMM, inner mitochondrial membrane; Mb, myoglobin; mtHSP70, mitochondrial heat shock protein 70; MTR, matrix; OMM, outer mitochondrial membrane; PK, proteinase K; Tom70, translocase of outer membrane 70.

We have previously shown that Mb is localized within mitochondria in rat skeletal muscle (Koma et al., 2021). To ascertain whether Mb is localized within the mitochondria in cultured skeletal muscle cells, we used the PK protection assay to remove proteins on the OMM of purified mitochondria while preserving the membrane structure (Boengler et al., 2009; Koma et al., 2021; Lechauve et al., 2012; Tammineni et al., 2013; van Vlies et al., 2007). We detected Mb and mitochondrial marker proteins in the purified mitochondria. Mb was detected in the untreated and PK‐treated mitochondria (Figure 2). Tom70, an OMM marker, could not be detected in PK‐treated mitochondria (Figure 2). AIF [inner mitochondrial membrane (IMM) marker] and mtHSP70 [matrix (MTR) marker] were detected in untreated and PK‐treated mitochondria (Figure 2). These data indicate that Mb is localized within the mitochondria of C2C12 myotubes. Because we demonstrated in rat skeletal muscle that Mb inside mitochondria is localized in the IMS (Koma et al., 2021), it is speculated that Mb inside mitochondria of C2C12 myotube is also localized in the IMS.

### 3.3 Mb interacts with TOM complex receptors (Tom20 and Tom70)

To pass through the TOM complex, it is first necessary to be recognized by the receptors. The TOM complex is mainly composed of the two import receptors Tom20 and Tom70. Tom20 recognizes the pre‐sequence of mitochondrial proteins, whereas Tom70 recognizes internal signal sequences of proteins (Schmidt et al., 2010; Wiedemann & Pfanner, 2017). Since Mb is not a mitochondrial protein, it is not certain whether it is recognized by Tom20 or Tom70. When investigating whether a protein is imported into the mitochondria via the TOM complex, the IP technique is initially used to ascertain whether a protein interacts with the receptors (Frank et al., 2015; Mackenzie et al., 2013; Rodriguez‐Sinovas et al., 2006; Santos & Kowluru, 2013). Therefore, we performed IP analysis of the mitochondrial fraction to determine if Mb is recognized by Tom20 and Tom70.

**FIGURE 3:**
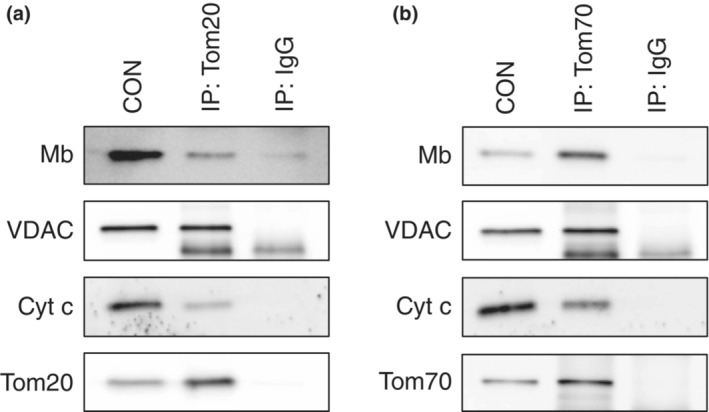
Mb interacts with TOM complex receptors, Tom20 and Tom70. IP analysis was performed using mitochondria isolated from C2C12 myotubes on differentiation day 5. Fifty micrograms of protein from the mitochondrial fraction were immunoprecipitated with anti‐Tom20, anti‐Tom70, or anti‐IgG rabbit antibody. Subsequently, immunoblotting was performed with anti‐Mb, anti‐VDAC, anti‐Cyt *c*, anti‐Tom20 (a), or anti‐Tom70 mouse antibody (b). In every assay, 2.5 μg of the mitochondrial fraction from C2C12 myotubes on differentiation day 5 was used as a positive control (CON). Immunoprecipitation of normal rabbit IgG was used as a negative control. Unprocessed blots are available in Figure S3. CON, control; Cyt *c*, cytochrome *c*; IgG, immunoglobulin G; IP, immunoprecipitation; Mb, myoglobin; Tom20, translocase of outer membrane 20; Tom70, translocase of outer membrane 70; VDAC; voltage‐dependent anion channel.

Since TOM complex is involved in the mitochondrial import of Cyt *c* and VDAC (Diekert et al., 2001; Kozjak‐Pavlovic et al., 2007), Cyt *c* and VDAC were used as positive controls for the IP analysis. Tom20 and Tom70 co‐precipitated with Mb, Cyt *c*, and VDAC in the mitochondrial fraction, whereas anti‐rabbit IgG produced only a faint precipitate with one of the analyzed protein fractions (Figure 3a,b). These data showed that Mb interacts with Tom20 and Tom70, suggesting the possibility that Mb is recognized by Tom20 or Tom70.

### 3.4 Individual knockdown of TOM complex receptors does not alter Mb expression in the mitochondrial fraction

Based on our previous results, we hypothesized that Mb passes through the TOM complex after being recognized by Tom20 or Tom70. If this hypothesis is correct, knockdown of Tom20 or Tom70 would reduce Mb expression in the mitochondrial fraction. Therefore, we tested the effect of individual knockdown of TOM complex receptors on mitochondrial import of Mb. Immunoblotting analysis was performed to evaluate mitochondrial proteins and Mb in the mitochondrial fraction in siRNA‐transfected myotubes.

Since Tom20 and Tom70 are involved in the mitochondrial import of COX‐IV and PINK1, respectively (Abe et al., 2000; Brix et al., 1999; Huang et al., 2012; Kato et al., 2013; Maruszczak et al., 2022), COX‐IV and PINK1 were used as positive controls for the Tom20‐ and Tom70‐dependent import pathways, respectively. Furthermore, AIF was used as negative control because AIF import into mitochondria is independent of the TOM complex (Chiang et al., 2012).

**FIGURE 4:**
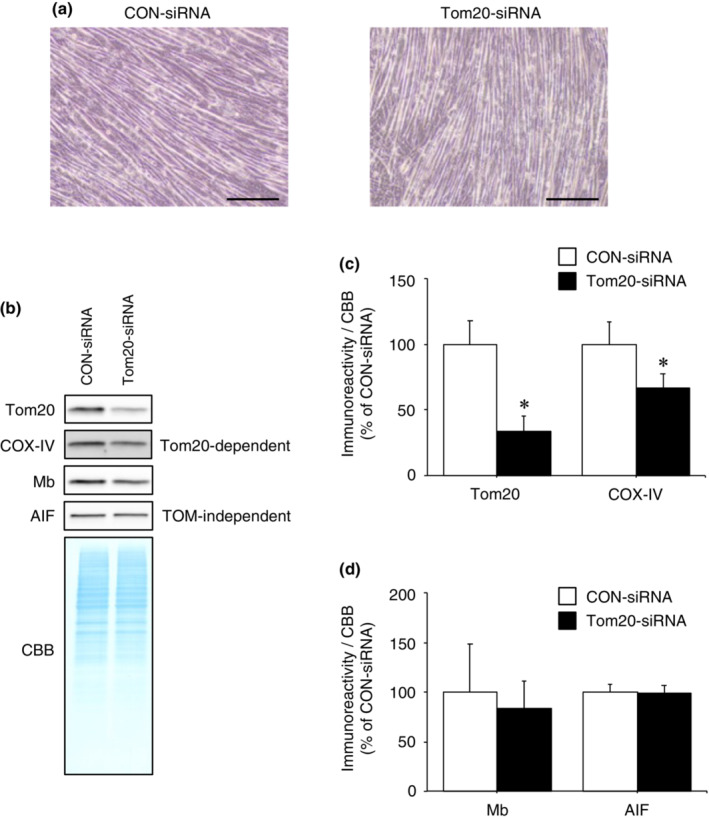
Mb is imported into mitochondria in the absence of Tom20. (a) CON‐siRNA or Tom20‐siRNA‐transfected myoblasts were differentiated into myotubes following incubation in differentiation medium for 3 days. Scale bar shows 200 μm. (b) Mitochondria were isolated from C2C12 myotubes transfected with Tom20‐siRNA or CON‐siRNA. Mitochondrial fractions were immunoblotted for Tom20, COX‐IV, Mb, and AIF. (c) Quantification of the immunoreactivities of siRNA‐target protein Tom20 and COX‐IV (Tom20‐dependent import) (*n* = 3/group). (d) Quantification of the immunoreactivities of Mb and AIF (TOM complex‐independent import) (*n* = 3/group). All calculated data were normalized to CBB staining. The mean immunoreactivity of proteins in CON‐siRNA‐transfected cells was set to 100%. Values are means ± standard deviation. Significant differences were assessed using an unpaired *t‐*test. * indicates significantly different from CON‐siRNA‐transfected cells (*p* < 0.05). Unprocessed blots are available in Figure S4. AIF, apoptosis‐inducing factor; CBB, Coomassie brilliant blue; CON, control; COX‐IV, cytochrome *c* oxidase subunit IV; Mb, myoglobin; TOM, translocase of the outer membrane; Tom20, translocase of outer membrane 20.

Following 3 days of differentiation (Figure 4a), Tom20 immunoreactivity in Tom20‐siRNA‐transfected cells was significantly decreased compared to CON‐siRNA‐transfected cells (Figure 4b,c; *p* < 0.05). Furthermore, immunoreactivity of COX‐IV (imported via Tom20‐dependent pathway) in Tom20‐siRNA‐transfected cells was significantly reduced compared to CON‐siRNA‐transfected cells (Figure 4b,c; *p* < 0.05). These results indicate that Tom20 knockdown was successful, and consequently reduced mitochondria protein import via the Tom20‐dependent pathway. However, immunoreactivities of Mb and AIF (imported via a TOM complex‐independent pathway) in Tom20‐siRNA‐transfected cells was not significantly different from CON‐siRNA‐transfected cells (Figure 4b,d). These data suggest that Tom20 recognition is not required for Mb import into mitochondria.

**FIGURE 5:**
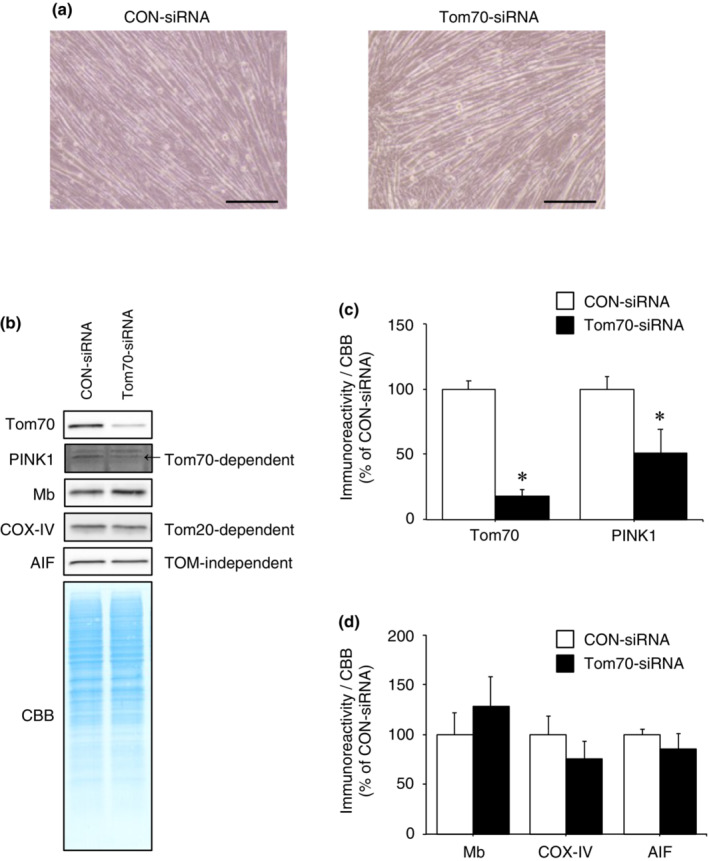
Mb is imported into mitochondria in the absence of Tom70. (a) CON‐siRNA or Tom70‐siRNA‐transfected myoblasts were differentiated into myotubes following incubation in differentiation medium for 3 days. Scale bar shows 200 μm. (b) Mitochondria were isolated from C2C12 myotubes transfected with Tom70‐siRNA or CON‐siRNA. Mitochondrial fractions were immunoblotted for Tom70, PINK1, Mb, and AIF. (c) Quantification of the immunoreactivities of siRNA‐target protein Tom70 and PINK1 (Tom70‐dependent import) (*n* = 3/group). (d) Quantification of the immunoreactivities of Mb, COX‐IV (Tom20‐dependent import) and AIF (TOM complex‐independent import) (*n* = 3/group). All calculated data were normalized to CBB staining. The mean immunoreactivity of proteins in CON‐siRNA‐transfected cells was set to 100%. Values are means ± standard deviation. Significant differences were assessed using an unpaired *t‐*test. * indicates significantly different from CON‐siRNA‐transfected cells (*p* < 0.05). Unprocessed blots are available in Figure S5. AIF, apoptosis‐inducing factor; CBB, Coomassie brilliant blue; CON, control; Mb, myoglobin; PINK1, PTEN‐induced kinase 1; TOM, translocase of the outer membrane; Tom70, translocase of outer membrane 70.

Three days after differentiation (Figure 5a), Tom70 immunoreactivity in Tom70‐siRNA‐transfected cells was significantly decreased compared to CON‐siRNA‐transfected cells (Figure 5b,c; *p* < 0.05). Furthermore, immunoreactivity of PINK1 (imported via a Tom70‐dependent pathway) in Tom70‐siRNA‐transfected cells was significantly reduced compared to CON‐siRNA‐transfected cells (Figure 5b,c: *p* < 0.05). These results indicate that Tom70 knockdown was successful, and consequently reduced mitochondrial protein import via the Tom70‐dependent pathway. However, immunoreactivities of Mb, COX‐IV (imported via Tom20‐dependent pathway), and AIF (imported via TOM complex‐independent pathway) in Tom70‐siRNA‐transfected cells was not significantly different from CON‐siRNA‐transfected cells (Figure 5b,d). These data suggest that Tom70 recognition is not required for Mb import into mitochondria.

### 3.5 Double knockdown of TOM complex receptors does not alter Mb expression in the mitochondrial fraction

**FIGURE 6:**
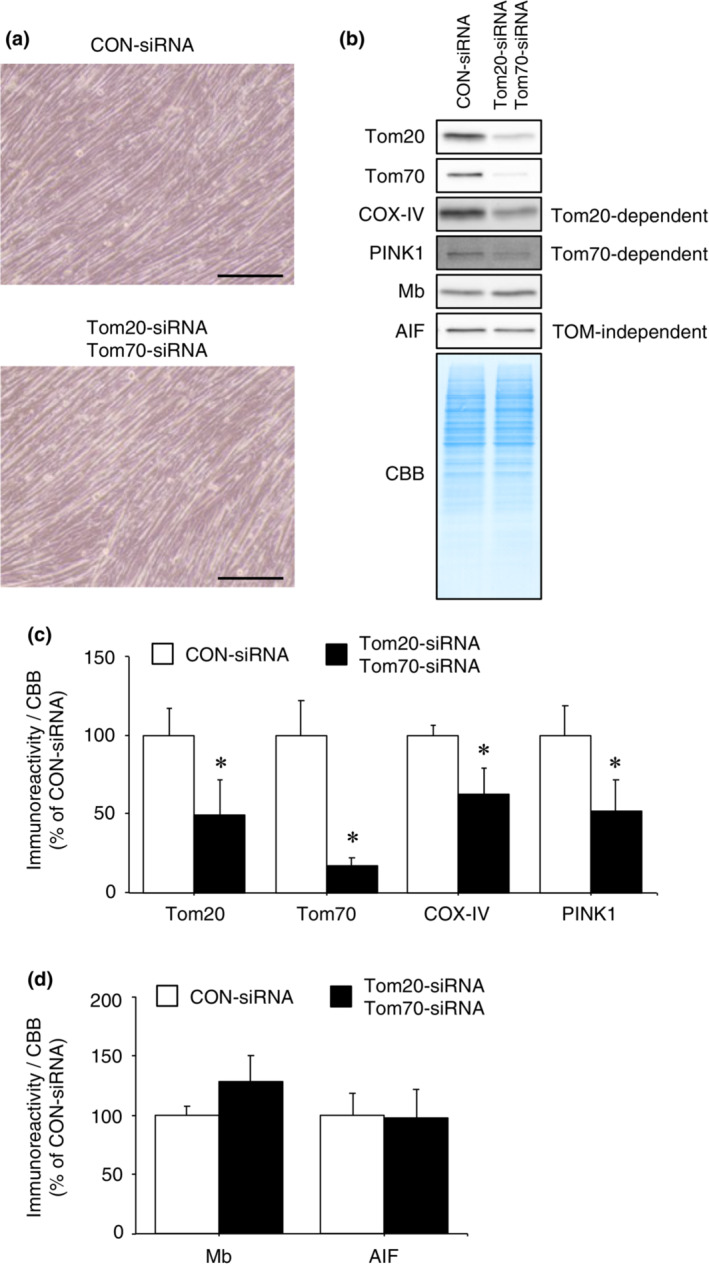
Mb is imported into mitochondria in the absence of Tom20 and Tom70. (a) CON‐siRNA or Tom20‐siRNA and Tom70‐siRNA double transfected myoblasts were differentiated into myotubes following incubation in differentiation medium for 3 days. Scale bar shows 200 μm. (b) Mitochondria were isolated from C2C12 myotubes transfected with Tom20‐siRNA and Tom70‐siRNA or CON‐siRNA. Mitochondrial fractions were immunoblotted for Tom20, Tom70, COX‐IV, PINK1, Mb, and AIF. (c) Quantification of the immunoreactivities of siRNA‐target protein Tom20 and Tom70, COX‐IV and PINK1 (Tom20‐ and Tom70‐dependent import, respectively) (*n* = 3/group). (d) Quantification of the immunoreactivities of Mb and AIF (TOM complex‐independent import) (*n* = 3/group). All calculated data were normalized to CBB staining. The mean immunoreactivity of proteins in CON‐siRNA‐transfected cells was set to 100%. Values are means ± standard deviation. Significant differences were assessed using an unpaired *t‐*test. * indicates significantly different from CON‐siRNA‐transfected cells (*p* < 0.05). Unprocessed blots are available in Figure S6. AIF, apoptosis‐inducing factor; CBB, Coomassie brilliant blue; CON, control; COX‐IV, cytochrome *c* oxidase subunit IV; Mb, myoglobin; PINK1, PTEN‐induced kinase 1; TOM, translocase of the outer membrane; Tom20, translocase of outer membrane 20; Tom70, translocase of outer membrane 70.

Because Tom20 and Tom70 functionally cooperate during mitochondrial protein import, individual knockdown of either receptor may not affect mitochondrial protein import (Hughes et al., 2016; Lithgow et al., 1994; Ramage et al., 1993). Therefore, we evaluated the effect of double knockdown of TOM complex receptors on mitochondrial import of Mb. After the experimental period (Figure 6a), Tom20 and Tom70 immunoreactivities in Tom20‐siRNA and Tom70‐siRNA double transfected cells were significantly decreased compared to CON‐siRNA‐transfected cells (Figure 6b,c; *p* < 0.05). Furthermore, immunoreactivities of COX‐IV (imported via Tom20‐dependent pathway) and PINK1 (imported via Tom70‐dependent pathway) in double transfected cells were significantly reduced compared to CON‐siRNA‐transfected cells (Figure 6b,c: *p* < 0.05). These results indicate that double knockdown of the two receptors was successful, and consequently reduced the mitochondria protein import via the Tom20‐ and Tom70‐dependent pathways. Nevertheless, immunoreactivities of Mb and AIF (imported via TOM complex‐independent pathway) in double transfected cells showed no significant differences compared with CON‐siRNA‐transfected cells (Figure 6b,d). These data indicate that Tom20 and Tom70 recognition is not required for Mb to be imported into mitochondria.

### 3.6 Knockdown of the TOM complex import pore does not alter Mb expression in the mitochondrial fraction

**FIGURE 7:**
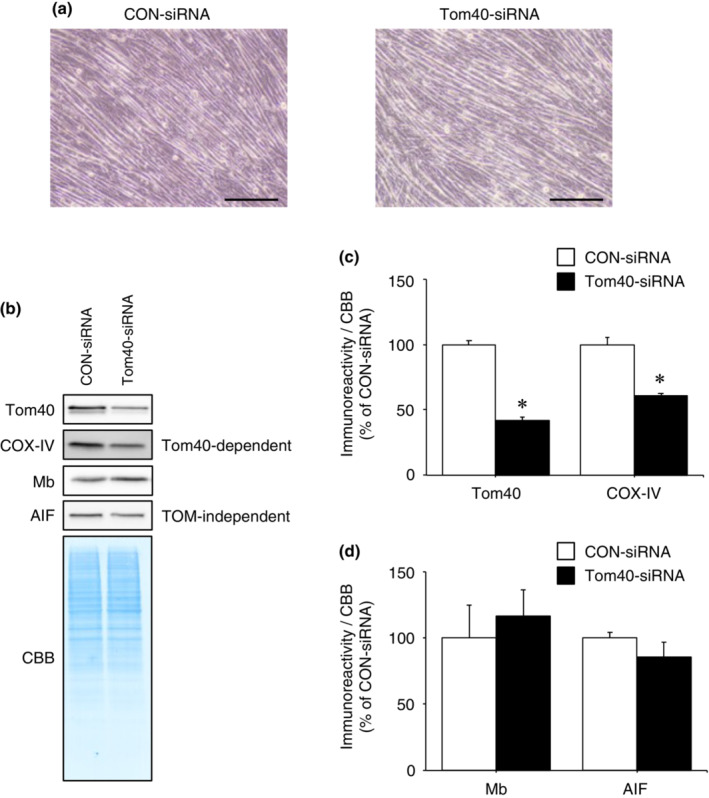
Mb is imported into mitochondria in the absence of Tom40. (a) CON‐siRNA or Tom40‐siRNA‐transfected myoblasts were differentiated into myotubes following incubation in differentiation medium for 3 days. Scale bar shows 200 μm. (b) Mitochondria were isolated from C2C12 myotubes transfected with Tom40‐siRNA or CON‐siRNA. Mitochondrial fractions were immunoblotted for Tom40, COX‐IV, Mb, and AIF. (c) Quantification of the immunoreactivities of siRNA‐target protein Tom40 and COX‐IV (Tom40‐dependent import) (*n* = 3/group). (d) Quantification of the immunoreactivities of Mb and AIF (TOM complex‐independent import) (*n* = 3/group). All calculated data were normalized to CBB staining. The mean immunoreactivity of proteins in CON‐siRNA‐transfected cells was set to 100%. Values are means ± standard deviation. Significant differences were assessed using an unpaired *t‐*test. * indicates significantly different from CON‐siRNA‐transfected cells (*p* < 0.05). Unprocessed blots are available in Figure S7. AIF, apoptosis‐inducing factor; CBB, Coomassie brilliant blue; CON, control; COX‐IV, cytochrome *c* oxidase subunit IV; Mb, myoglobin; TOM, translocase of the outer membrane; Tom40, translocase of outer membrane 40.

Tom40 is the only TOM complex import pore. To exclude the possibility that Mb passes through the TOM complex without recognition by its receptors, we tested the effect of knockdown of the TOM complex import pore (Tom40) on mitochondrial import of Mb. Since Tom40 is involved in the mitochondrial import of complex I and complex IV proteins (Bender et al., 2013; Frank et al., 2015; Huang et al., 2012), ubiquinone oxidoreductase subunit B8 (NDUFB8; complex I subunit) or COX‐IV (complex IV subunit) was used as positive control for Tom40‐dependent import pathway. After the differentiation (Figure 7a), Tom40 immunoreactivity in Tom40‐siRNA‐transfected cells was significantly decreased compared with CON‐siRNA‐transfected cells (Figure 7b,c; *p* < 0.05). Furthermore, immunoreactivity of COX‐IV (imported via Tom40‐dependent pathway) in Tom40‐siRNA‐transfected cells was significantly reduced compared with CON‐siRNA‐transfected cells (Figure 7b,c; *p* < 0.05). These results indicate that knockdown of Tom40 was successful, and consequently reduced mitochondria protein import via the Tom40‐dependent pathway. However, immunoreactivities of Mb and AIF (imported via TOM complex‐independent pathway) in Tom40‐siRNA‐transfected cells were not significantly different compared to CON‐siRNA‐transfected cells (Figure 7b,d).

**FIGURE 8:**
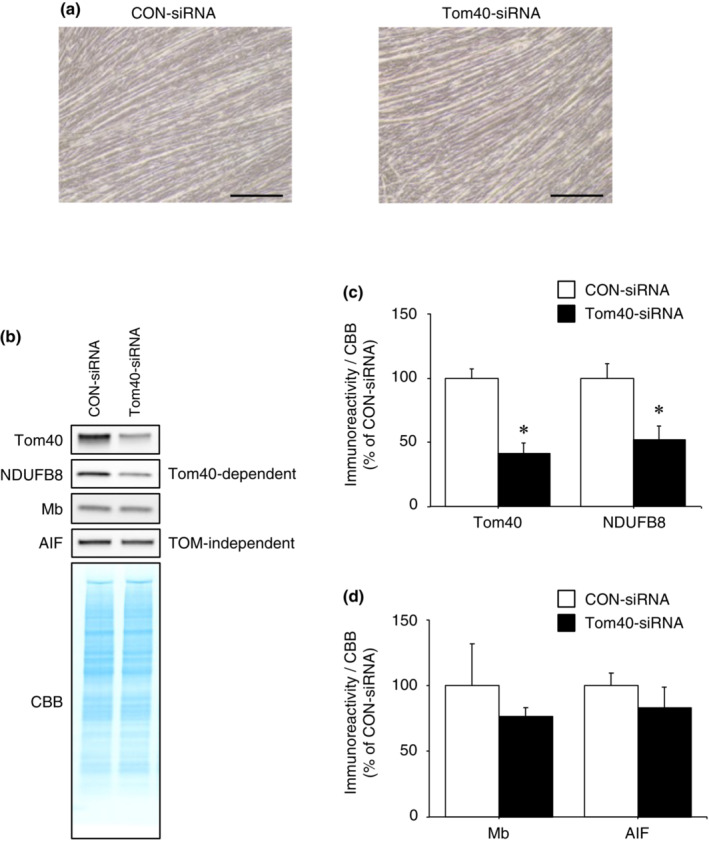
Tom40 is not involved in Mb import into mitochondria during differentiation until day 5 in C2C12 cells. (a) Images of CON‐siRNA or Tom40‐siRNA‐transfected myotubes on day 5 of differentiation. Scale bar shows 200 μm. (b) Mitochondria were isolated from C2C12 myotubes transfected with Tom40‐siRNA or CON‐siRNA. Mitochondrial fractions were immunoblotted for Tom40, NDUFB8, Mb, and AIF. (c) Quantification of the immunoreactivities of siRNA‐target protein Tom40 and NDUFB8 (Tom40‐dependent import) (*n* = 3/group). (d) Quantification of the immunoreactivities of Mb and AIF (TOM complex‐independent import) (*n* = 3/group). All calculated data were normalized to CBB staining. The mean immunoreactivity of proteins in CON‐siRNA‐transfected cells was set to 100%. Values are means ± standard deviation. Significant differences were assessed using an unpaired *t‐*test. * indicates significantly different from CON‐siRNA‐transfected cells (*p* < 0.05). Unprocessed blots are available in Figure S8. AIF, apoptosis‐inducing factor; CBB, Coomassie brilliant blue; CON, control; Mb, myoglobin; NDUFB8, ubiquinone oxidoreductase subunit B8; TOM, translocase of the outer membrane; Tom40, translocase of outer membrane 40.

Since Mb expression in C2C12 myotube cells on day 3 of differentiation is small (Ordway & Garry, 2004), we cannot fully deny the possibility that few TOM complexes remaining after the siRNA transfection was sufficient to transport Mb into mitochondria. We confirmed that Mb expression in the cytosolic and mitochondrial fractions of myotubes on day 5 of differentiation was approximately 60%–70% higher than that on day 3 of differentiation (data not shown). Therefore, to exclude the above possibility, we also investigated the effect of Tom40 knockdown on mitochondrial import of Mb in myotubes on day 5 of differentiation. Following 5 days of differentiation (Figure 8a), Tom40 immunoreactivity in Tom40‐siRNA‐transfected cells was significantly decreased compared with CON‐siRNA‐transfected cells (Figure 8b,c; *p* < 0.05). Furthermore, immunoreactivity of NDUFB8 (imported via Tom40‐dependent pathway) in Tom40‐siRNA‐transfected cells was significantly reduced compared with CON‐siRNA‐transfected cells (Figure 8b,c; *p* < 0.05). Immunoreactivities of Mb and AIF (imported via TOM complex‐independent pathway) in Tom40‐siRNA‐transfected cells were not significantly different compared to CON‐siRNA‐transfected cells (Figure 8b,d). These results suggest that the import of Mb into mitochondria does not necessarily require the TOM complex at least during the differentiation of skeletal muscle cells.

## 4 DISCUSSION

In our previous studies, we found that the O_2_‐binding protein Mb is not only localized in cytosol but also within mitochondrial IMS in skeletal muscle (Koma et al., 2021; Yamada et al., 2013). We have also confirmed that Mb‐overexpression in C2C12 myotubes results in improved mitochondrial respiration via augmentation of complex IV activity (Yamada et al., 2016), that led to the supposition that Mb inside mitochondria may upregulate mitochondrial respiratory function. Although the presence of Mb within the mitochondria means that there is a system by which Mb is imported into the mitochondria, the mechanism remains unknown. Since most IMS proteins are translocated through the TOM complex across the OMM (Kulawiak et al., 2013), the present study focused on the TOM complex to elucidate the mechanism of Mb import into the mitochondria of myocytes. Although we found that Mb interacts with TOM complex receptors (Tom20, Tom70), knockdown of TOM complex subunits showed no clear effects on Mb expression in the mitochondrial fraction, suggesting that Mb import does not necessarily require the TOM complex. We speculate that there is a TOM complex‐independent mechanism responsible for Mb import into mitochondria.

Mb levels in the mitochondrial fraction are lower than in the cytosol (Yamada et al., 2013). Thus, isolation of a pure mitochondrial fraction without contamination with cytosolic proteins from skeletal muscle and cells is necessary to accurately verify Mb expression in the mitochondria. We have previously shown that our isolation method is capable of isolating pure mitochondria from rat skeletal muscle, but we have not verified that the method is equally applicable for cultured skeletal muscle cells (Koma et al., 2021). In the present study, we confirmed that our isolation method completely separated the mitochondrial and cytosolic fractions of C2C12 myotubes. This observation excluded the possibility that cytosolic proteins, including cytosolic Mb, contaminate the mitochondrial fraction studied herein. Thus, it appears that our isolation method is capable of isolating pure mitochondria from both skeletal muscle tissue and cultured skeletal muscle cells (Koma et al., 2021).

Traditionally, Mb has been viewed as a cytosolic O_2_‐binding protein (Kanatous & Mammen, 2010; Ordway & Garry, 2004; Postnikova & Shekhovtsova, 2018). Although we recently showed that Mb is localized within the mitochondria in rat skeletal muscle using the PK protection assay (Koma et al., 2021), it remains unknown whether Mb is localized within the mitochondria in cultured skeletal muscle cells, such as C2C12 myotubes. Since the localization of certain proteins, such as Stat3, within mitochondria depends on the cell type or species (Su et al., 2020; Tammineni et al., 2013; Wegrzyn et al., 2009), it was necessary to confirm whether Mb localizes in mitochondria of skeletal muscle cells derived from mice (C2C12 myotubes). In the present study, we demonstrated that Mb is localized within the mitochondria of cultured mouse muscle cells. The present data are essential to understanding the mechanism of mitochondrial Mb import and suggest the possibility that Mb is universally present within mitochondria of rodent skeletal muscle.

The mitochondrial import system is composed of protein complexes in the OMM and IMM. The TOM complex serves as a translocator in the OMM, transporting proteins from the cytoplasm to the IMS (Becker et al., 2019; Wiedemann & Pfanner, 2017). Indeed, most proteins present in the IMS are translocated through the TOM complex across the OMM (Kulawiak et al., 2013). Since mitochondrial Mb is localized in the IMS in rat skeletal muscle (Koma et al., 2021), the mitochondrial import pathway for Mb may depend on the TOM complex. To pass through the TOM complex, it is first necessary to be recognized by the receptors. The TOM complex is mainly composed of two import receptors—Tom20 and Tom70—and the pore‐forming protein Tom40. Generally, Tom20 recognizes the pre‐sequence of mitochondrial proteins, whereas Tom70 recognizes internal signal sequences of proteins (Schmidt et al., 2010; Wiedemann & Pfanner, 2017). In the present study, we confirmed that Mb interacted with Tom20 and Tom70 in mitochondrial fractions. Since Tom20 interacts with Tom70 (Fan et al., 2011), it is logical that Mb was detected following IP with either Tom20 or Tom70. Furthermore, several previous studies have suggested that interactions with TOM complex receptors reflect the involvement of the TOM complex in the mitochondrial import of a protein (Mackenzie et al., 2013; Rodriguez‐Sinovas et al., 2006; Santos & Kowluru, 2013). Accordingly, we hypothesized that Mb utilizes the TOM complex to enter mitochondria.

Several previous studies have performed knockdown experiments on TOM complex subunits to investigate whether a protein is imported into mitochondria via a TOM complex‐dependent pathway (Chiang et al., 2012; Frank et al., 2015; Fu et al., 2012; Huang et al., 2012). Therefore, we examined the effect of knockdown of TOM complex subunits on mitochondrial import of Mb to test the hypothesis that the TOM complex is necessary for mitochondrial import of Mb. Since Mb expression is completely quiescent in myoblasts but increases during myotube differentiation (Kanatous & Mammen, 2010; Yamada et al., 2016), a C2C12 myotube transient transfection model, based on Chen et al. (2019), was established by myoblast transfection with siRNA of TOM complex subunits followed by induction of differentiation. Cells are typically harvested 24–72 h after transfection, and we confirmed that Mb was detected in the mitochondrial fractions obtained from myotubes on differentiation day 3 (data not shown). Thus, siRNA‐transfected myotubes on differentiation day 3 were used in the knockdown experiments. Contrary to expectations, the knockdown experiments for TOM complex receptors (Tom20, Tom70) and TOM complex channel (Tom40) did not alter the amount of Mb expression in the mitochondrial fraction. However, Mb expression in the cytosolic and mitochondrial fractions of C2C12 myotubes was approximately 60%–70% lower on day 3 than on day 5 of differentiation (data not shown). Therefore, the induction of Tom40 knockdown for 5 days after the differentiation of myotubes was performed to reject the possibility that the effect of knockdown of TOM complex on inhibiting mitochondrial import of Mb might not be substantial. As the result, the knockdown experiment also did not alter the amount of Mb expression in the mitochondrial fraction. Frank et al. (2015) have reported that the protein expression of Bim, which is an OMM‐integrated protein, in HeLa mitochondria did not significantly change following knockdown of TOM complex subunits (Tom20, Tom70, or Tom40) despite IP experiments showing a clear interaction between Bim and either Tom20 or Tom70. Based on their results, the authors concluded that the TOM complex is not essential for mitochondrial import of the Bim protein (Frank et al., 2015). Similar to the study of Frank et al. (2015), the results of the present study involving knockdown experiments suggest that the import of Mb into mitochondria does not necessarily require the TOM complex at least during the differentiation of skeletal muscle cells. However, we could not explain the physiological role of Mb‐TOM complex receptor interactions in the present study, which requires further studies to fully elucidate.

The present study suggests that import of Mb into mitochondria does not necessarily require the TOM complex. The mitochondrial permeability transition pore (mPTP) is responsible for metabolite exchange between the cytosol and the mitochondrial MTR (Šileikytė & Forte, 2019). Several previous studies have reported that certain proteins localized in mitochondrial MTR, such as p53 and neuroglobin, are translocated into mitochondria via the mPTP (Achanta et al., 2005; Lechauve et al., 2012; Liu et al., 2008; Yu et al., 2012). However, since we could not confirm the MTR localization of Mb (Koma et al., 2021), there is a low possibility that mPTP is involved in Mb import into mitochondria. Interestingly, it has been reported that AIF, which is an IMM protein that projects into the IMS, is fully translocated into HeLa mitochondria without the TOM complex (Chiang et al., 2012). It was shown in a previous study that AIF is imported from the endoplasmic reticulum into the mitochondria via transport vesicles during the mitochondrial fusion/fission process (Chiang et al., 2012). Since submitochondrial localization of Mb is proximal to AIF, Mb may be imported into mitochondria through the same transport vesicle pathway as AIF. Further studies are needed to examine whether Mb is localized in the endoplasmic reticulum and inhibition of mitochondrial fusion/fission impairs mitochondrial Mb import.

### 4.1 Limitations

In the present study, we did not investigate the effect of TOM complex overexpression on mitochondrial import of Mb. Therefore, we cannot fully rule out the possibility that TOM complex may play a role in promoting Mb transport into mitochondria when the number of TOM complex increases. To clarify this point, further study is necessary to determine the effect of increased TOM complex levels on mitochondrial Mb import. Furthermore, we did not investigate the effect of TOM complex knockdown on activities of mitochondrial enzymes and electron transport chain complexes because it was beyond the scope of this study. However, biochemical activity assay will also provide more detail on the effect of TOM complex knockdown on mitochondrial protein import. Therefore, this will be an issue for future study.

## 5 CONCLUSION

The present study showed that Mb is localized within the mitochondria in mouse C2C12 myotubes. Although we found that Mb interacts with TOM complex receptors (Tom20 and Tom70), knockdown experiments showed that the TOM complex is not essential for mitochondrial import of Mb at least during the differentiation of skeletal muscle cells. The present findings suggest that Mb utilizes a TOM‐independent mechanism to enter the mitochondria. The results described herein present a basis upon which to elucidate the mechanism of Mb import into mitochondria. Further studies are needed to clarify the physiological role of Mb‐TOM complex receptor interactions and to discover the alternative mitochondrial Mb import pathway.

## AUTHOR CONTRIBUTIONS

R.K., Y.A., T.Y., T.J., and K.M. contributes to the conception and design of this study. R.K., T.S., and Y.N. contributes to the data collection and analysis. R.K., T.S., Y.A., T.Y., and K.M. contributes to the data interpretation. R.K., T.S., Y.A., T.Y., T.J., Y.N., and K.M. contributes to the drafting and critical version of the manuscript. All authors have approved the final version of the manuscript and agree to be accountable for all aspects of the study. All person designated as authors qualify for authorship, and all those who qualify for authorship are listed.

## FUNDING INFORMATION

This research was supported by JSPS KAKENHI (21H03318, K.M.), JST SPRING (JPMJSP2135, R.K.), and Sasakawa Scientific Research Grant from The Japan Science Society (R.K.).

## CONFLICT OF INTEREST STATEMENT

The authors have no conflicts of interest to declare.

## Supporting information


Figure S1.

Figure S2.

Figure S3.

Figure S4.

Figure S5.

Figure S6.

Figure S7.

Figure S8.
