# Supplementary figures and images for: TOM complex‐independent transport pathway of myoglobin into mitochondria in C2C12 myotubes
Source: Physiol Rep. 2023 Apr 5;11(7):e15632. doi: 10.14814/phy2.15632 (PMC10076690; doi:10.14814/phy2.15632)

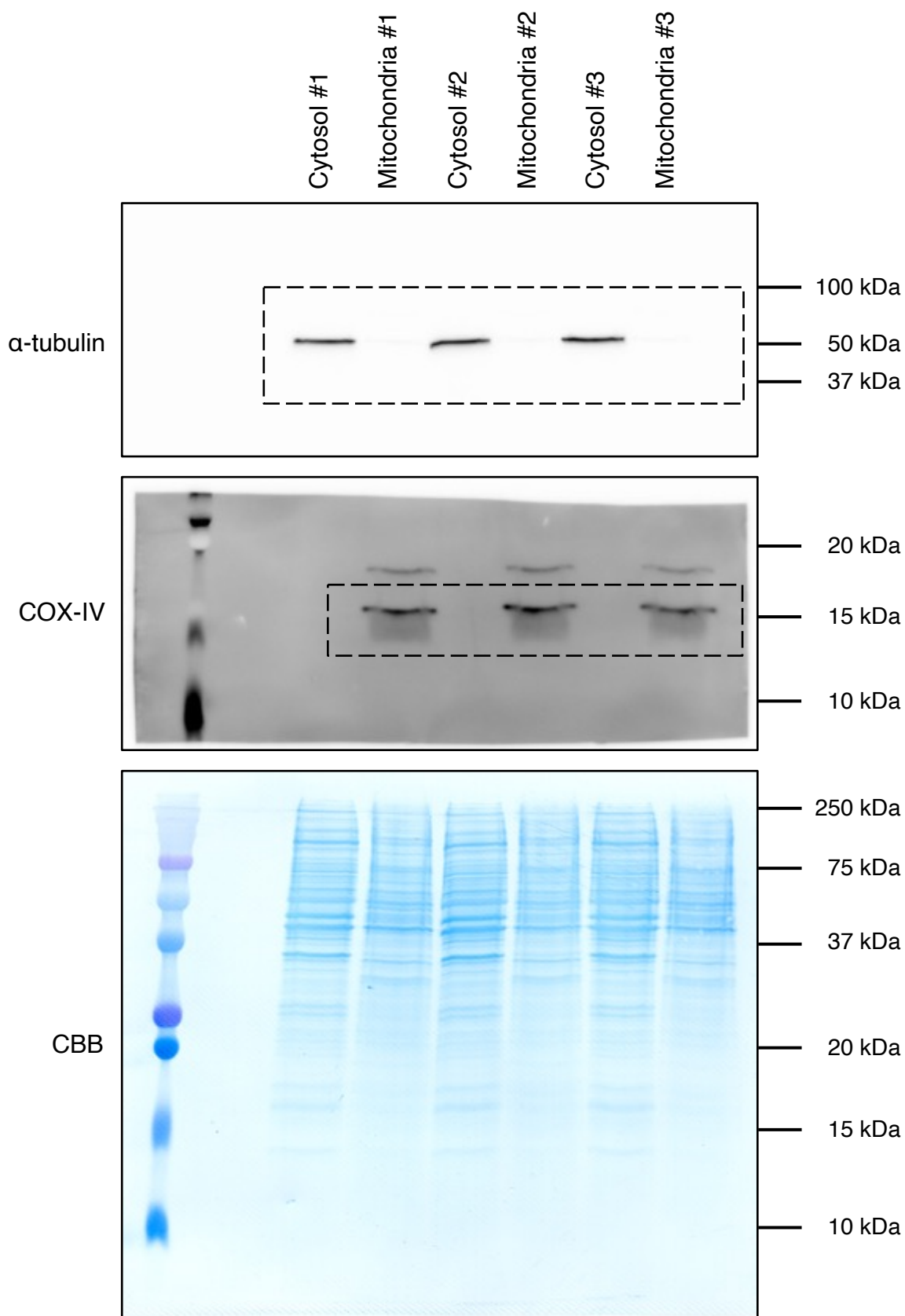

Supplemental Figure 1  
Koma et al. 2022

Supplement: Supplementary file 1 — Figure S1. [file PHY2-11-e15632-s007.pdf]

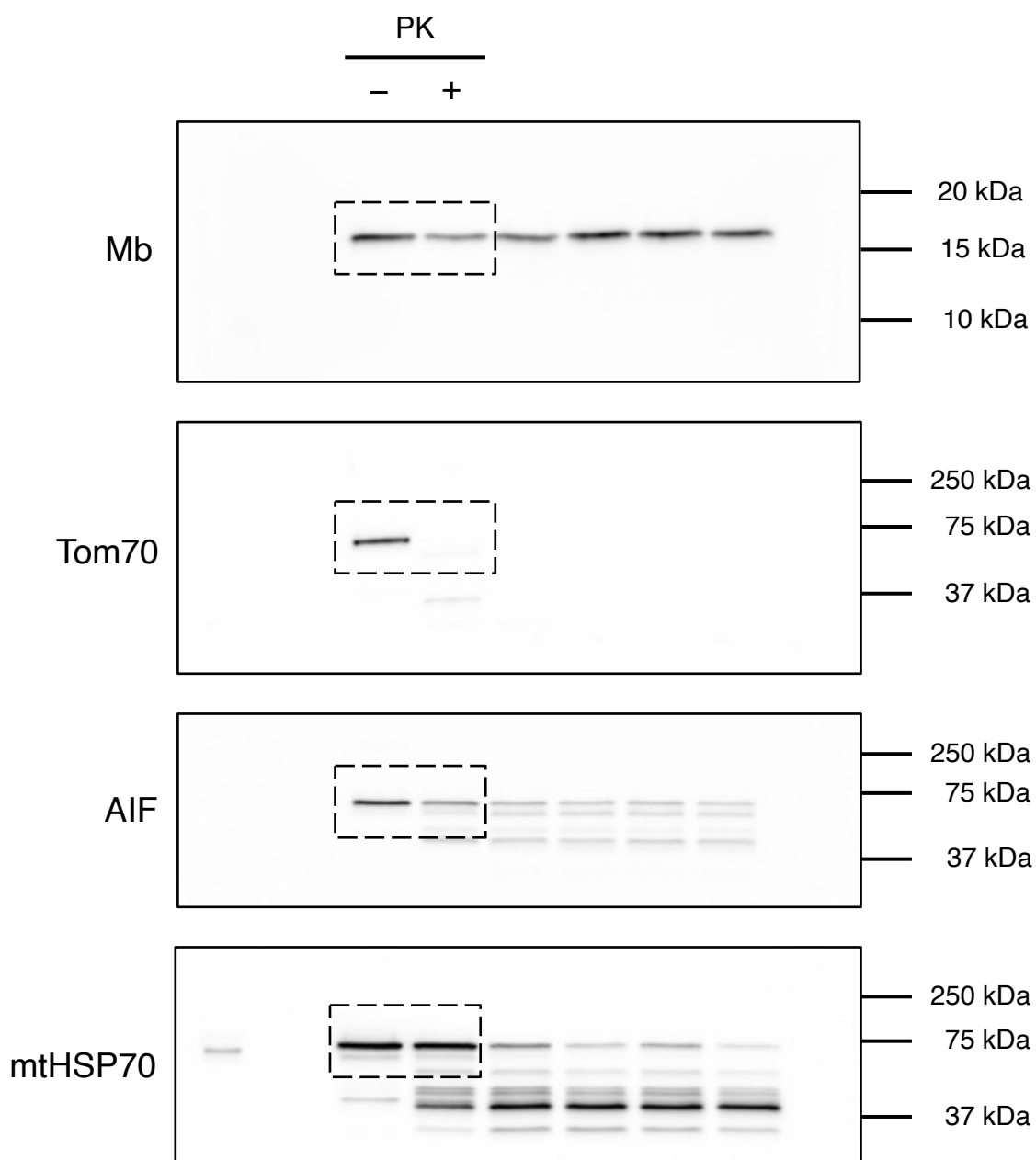

Supplemental Figure 2  
Koma et al. 2022

Supplement: Supplementary file 2 — Figure S2. [file PHY2-11-e15632-s003.pdf]

**A**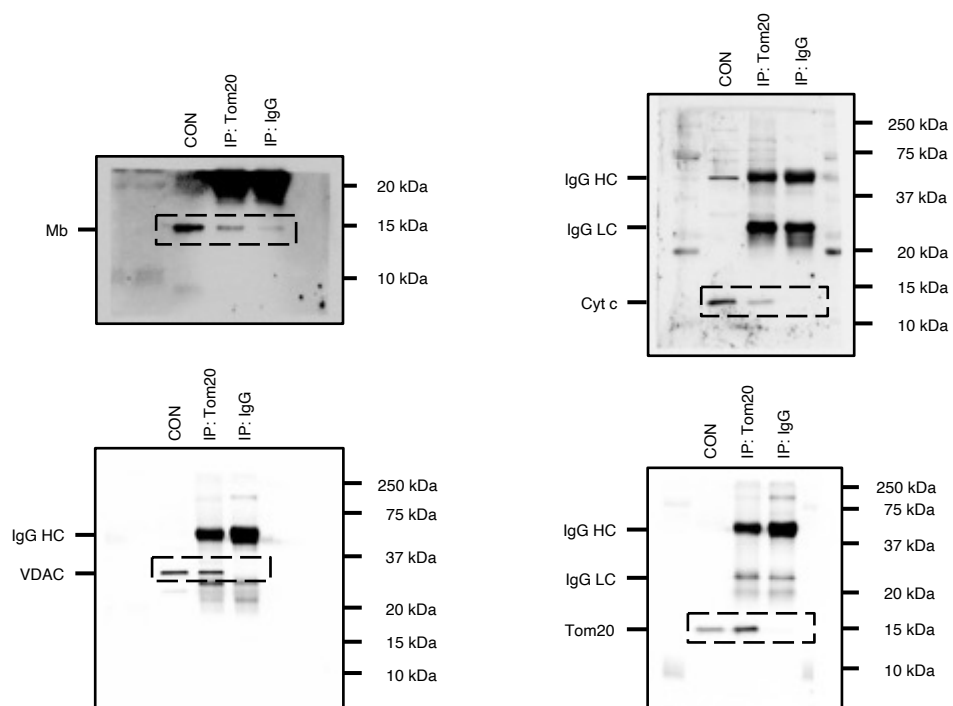**B**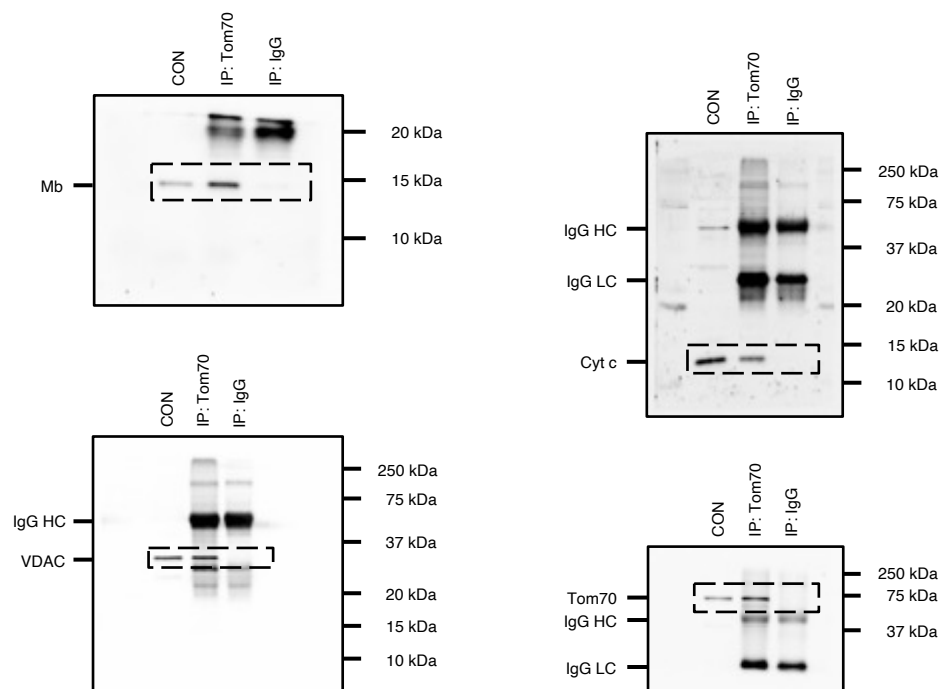

Supplement: Supplementary file 3 — Figure S3. [file PHY2-11-e15632-s008.pdf]

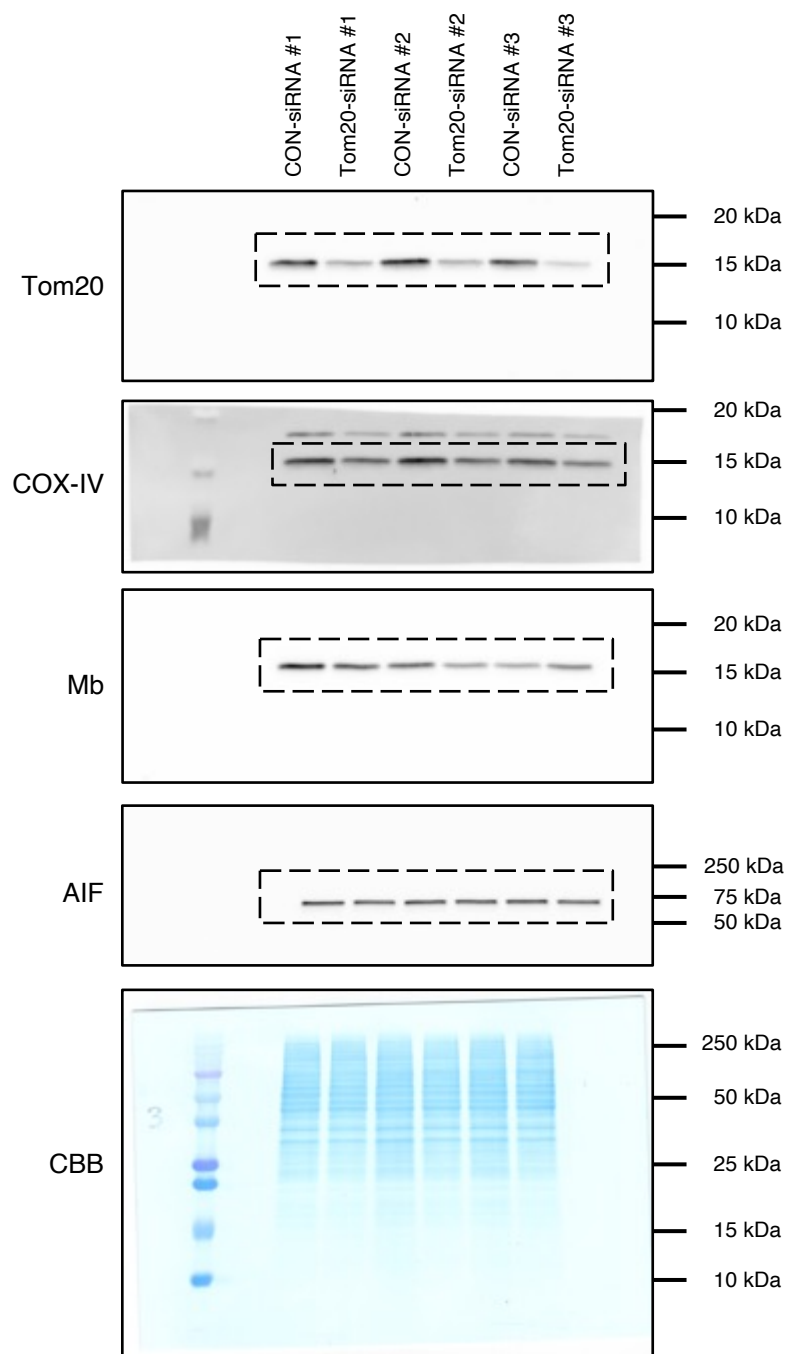

Supplemental Figure 4  
Koma et al. 2022

Supplement: Supplementary file 4 — Figure S4. [file PHY2-11-e15632-s004.pdf]

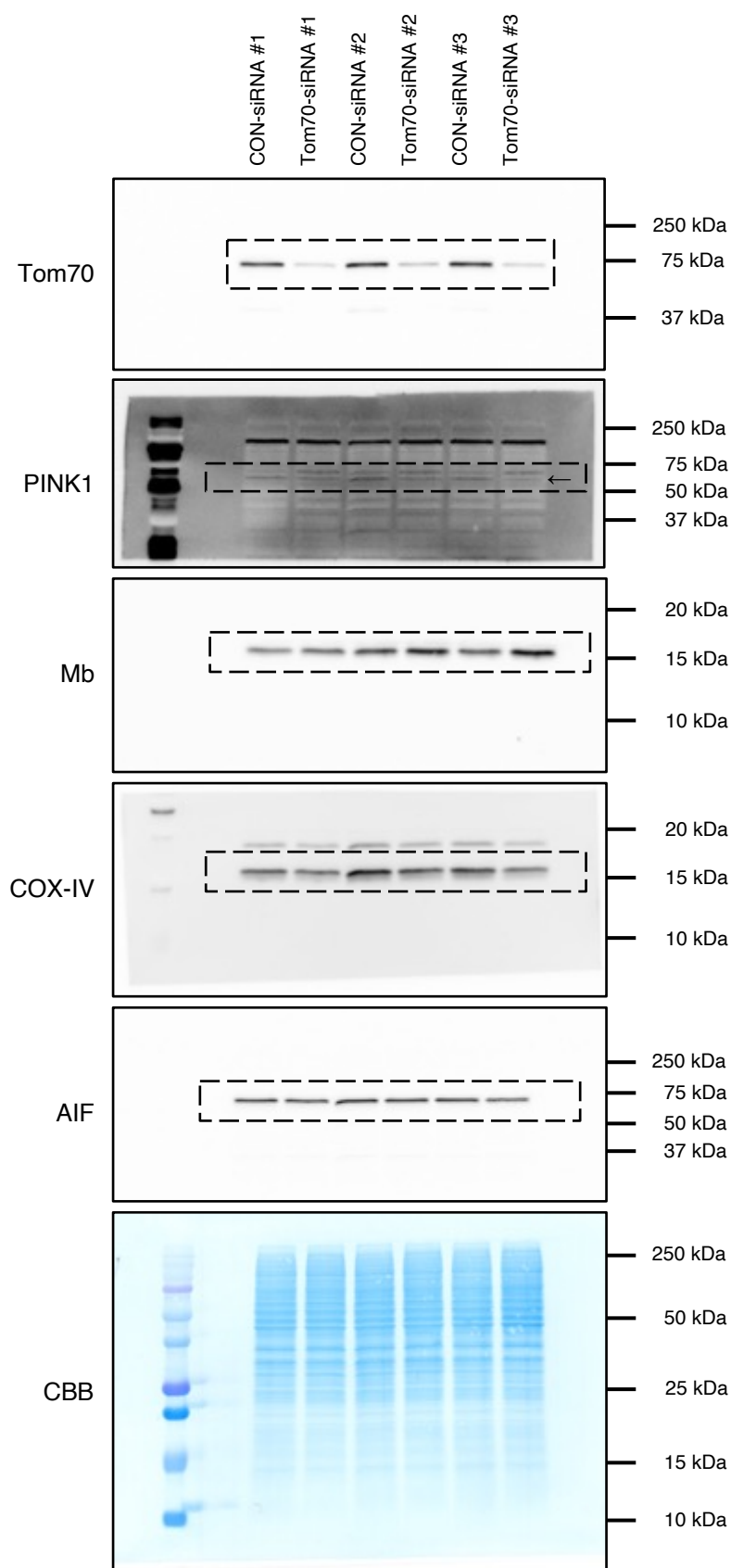

Supplemental Figure 5  
Koma et al. 2022

Supplement: Supplementary file 5 — Figure S5. [file PHY2-11-e15632-s005.pdf]

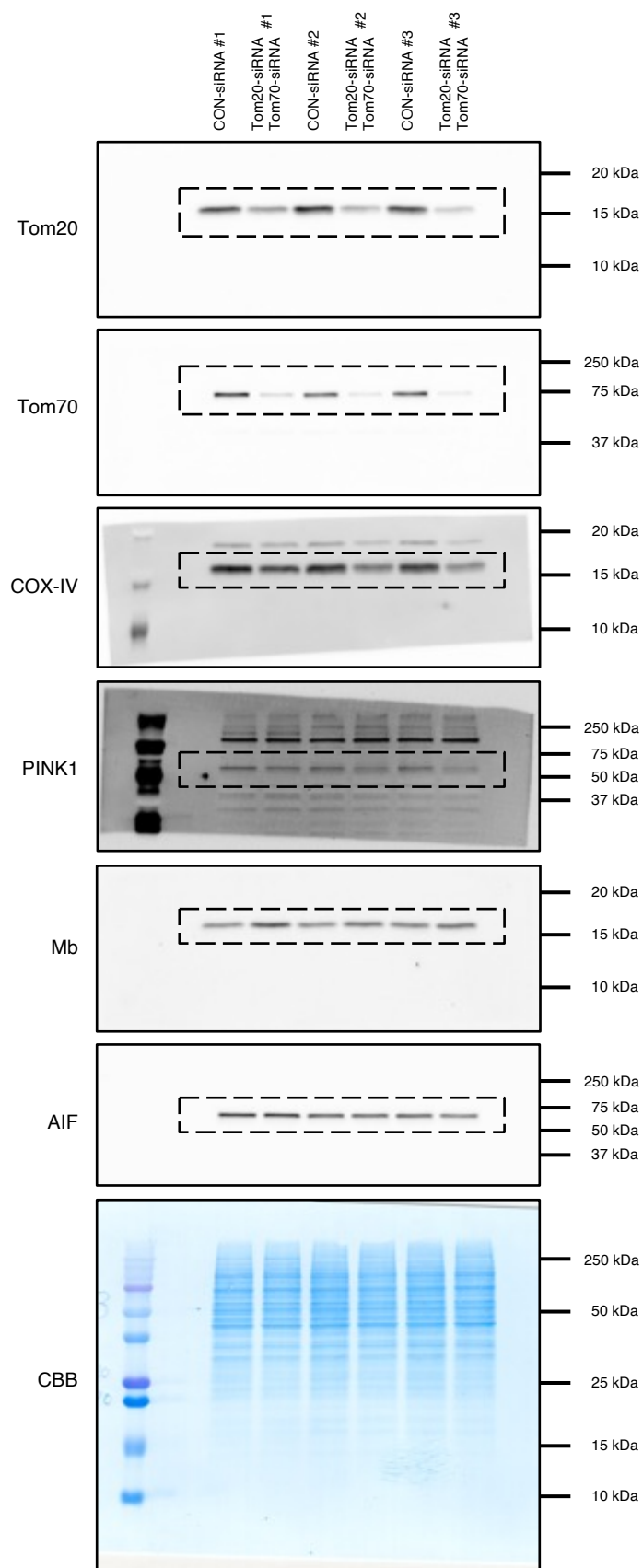

Supplemental Figure 6  
Koma et al. 2022

Supplement: Supplementary file 6 — Figure S6. [file PHY2-11-e15632-s006.pdf]

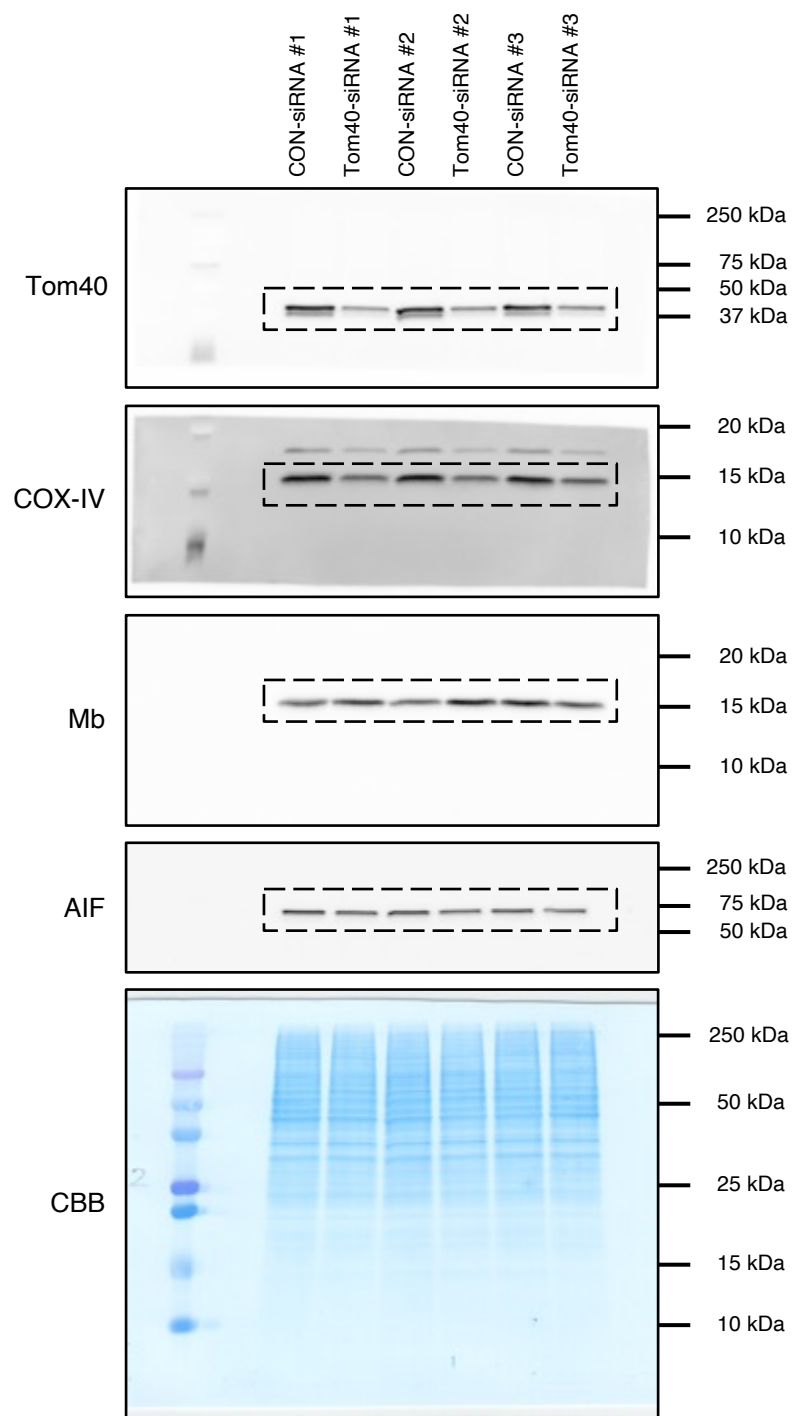

Supplemental Figure 7  
Koma et al. 2022

Supplement: Supplementary file 7 — Figure S7. [file PHY2-11-e15632-s001.pdf]

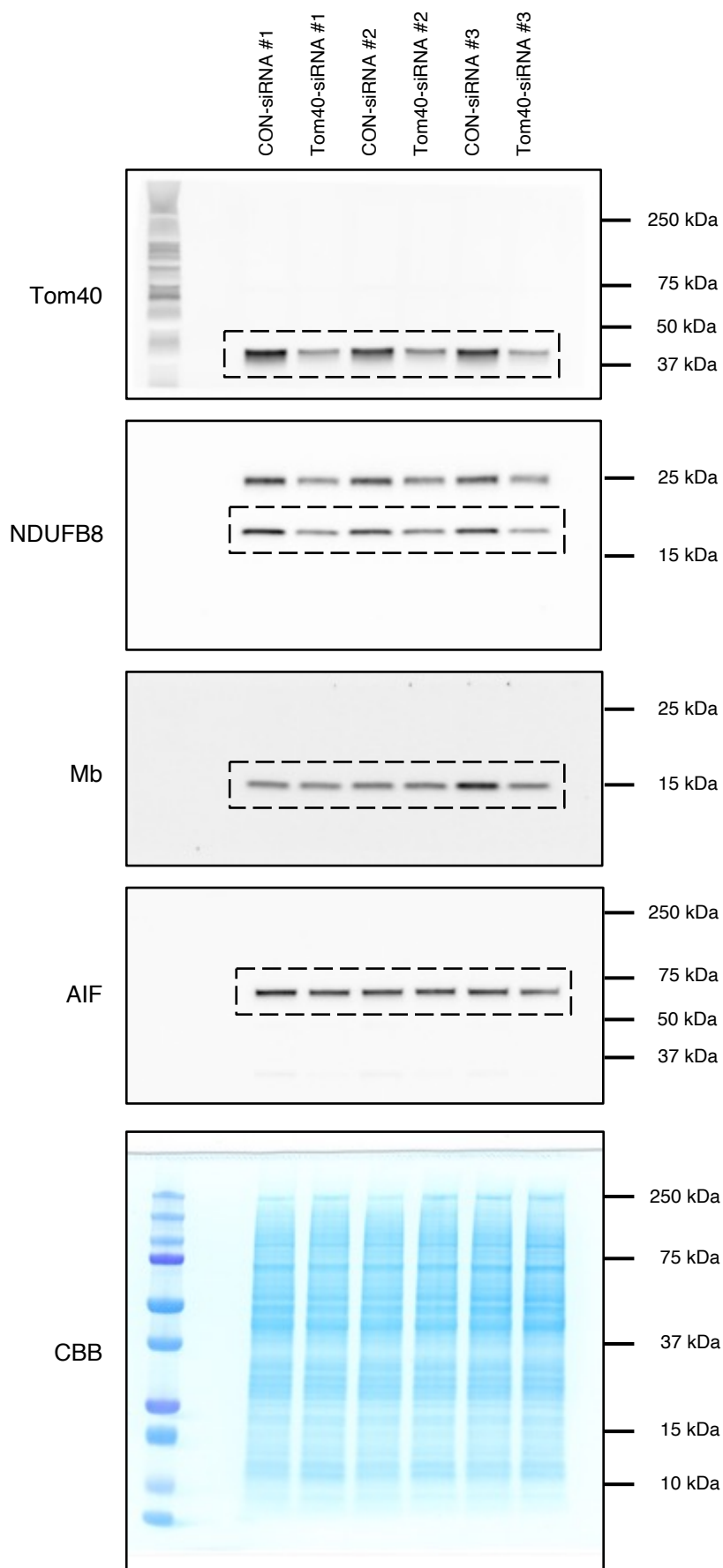

Supplemental Figure 8  
Koma et al. 2022

Supplement: Supplementary file 8 — Figure S8. [file PHY2-11-e15632-s002.pdf]
